# Current Concepts in Penetrating and Blast Injury to the Central Nervous System

**DOI:** 10.1007/s00268-014-2874-7

**Published:** 2014-12-02

**Authors:** Jeffrey V. Rosenfeld, Randy S. Bell, Rocco Armonda

**Affiliations:** 1 Department of Neurosurgery, The Alfred Hospital, Melbourne, Australia; 2 Department of Surgery, Monash University, 6th Floor, The Alfred Centre, 99 Commercial Road, Clayton, Melbourne, Australia; 3 Department of Surgery, F. Edward Hébert School of Medicine, Uniformed Services University of the Health Sciences, Bethesda, MD USA; 4 Department of Neurosurgery, MedStar-Georgetown University Hospital and Washington Hospital Center, Washington, DC USA

## Abstract

**Aim:**

To review the current management, prognostic factors and outcomes of penetrating and blast injuries to the central nervous system and highlight the differences between gunshot wound, blast injury and stabbing.

**Methods:**

A review of the current literature was performed.

**Results:**

Of patients with craniocerebral GSW, 66–90 % die before reaching hospital. Of those who are admitted to hospital, up to 51 % survive. The patient age, GCS, pupil size and reaction, ballistics and CT features are important factors in the decision to operate and in prognostication. Blast injury to the brain is a component of multisystem polytrauma and has become a common injury encountered in war zones and following urban terrorist events. GSW to the spine account for 13–17 % of all gunshot injuries.

**Conclusions:**

Urgent resuscitation, correction of coagulopathy and early surgery with wide cranial decompression may improve the outcome in selected patients with severe craniocerebral GSW. More limited surgery is undertaken for focal brain injury due to GSW. A non-operative approach may be taken if the clinical status is very poor (GCS 3, fixed dilated pupils) or GCS 4–5 with adverse CT findings or where there is a high likelihood of death or poor outcome. Civilian spinal GSWs are usually stable neurologically and biomechanically and do not require exploration. The indications for exploration are as follows: (1) compressive lesions with partial spinal cord or cauda equina injury, (2) mechanical instability and (3) complications. The principles of management of blast injury to the head and spine are the same as for GSW. Multidisciplinary specialist management is required for these complex injuries.

## Introduction

The majority of homicides and suicides involve the use of firearms and disproportionately affect persons <55 years, males and certain minority populations [1]. The experience of civilian neurosurgeons with penetrating CNS trauma varies depending on their location. CNS blast injuries have been encountered frequently by military surgeons in Iraq and Afghanistan and are increasingly encountered by civilian neurosurgeons because of terrorist bombings in urban environments. Craniocerebral gunshot wounds (GSWs) and blast-injured patients are arguably among the most complex and surgically challenging trauma encountered by neurosurgeons.

This review focuses on current concepts and treatment strategies for penetrating craniocerebral and spinal injury due to gunshot wounds (GSWs), blast injury and knives and other sharp implements. Aggressive management is usually recommended for craniocerebral GSW presenting with Glasgow Coma Score (GCS) 6–12. There is controversy as to how aggressively to treat the craniocerebral GSW in patients with GCS 3–5 [2]. Many authors have advocated an expectant approach because of likely poor outcome or death. However, there has been recent advocacy for a more ‘aggressive’ approach to the management of these patients with evidence emerging of improved survivability. For instance, in one trauma center in Tucson, Arizona, survival rates improved from 10 % in 2008 to 46 % in 2011 [3]. In this review, we describe the likely factors which have resulted in the improved outcomes for these patients and discuss selection of patients for surgery, the principles of surgery and prognosis.

The treatment of penetrating spine injuries remains controversial due to the weak strength of the evidence for different treatment strategies [4]. Military penetrating spinal injury tends to be more destructive than civilian injury and may require more extensive surgery. Civilian neurosurgeons on call for trauma should be prepared to manage penetrating CNS trauma.

This review is based on the current literature, published guidelines and our personal experience of managing patients with penetrating and blast injury to the CNS and does not represent the official policy of either United States Department of Defense or the Australian Defence Force.

## Craniocerebral GSW

### Epidemiology

Craniocerebral GSWs are the most lethal of civilian firearm injuries with up to 71 % dying at the scene, 66–90 % dying before reaching hospital and survivals of up to 51 % reported of those reaching hospital [3, 5, 6]. The mortality is higher with self-inflicted GSW because of the close range of the weapon. In a recent study of craniocerebral GSW in the State of Maryland, USA, there were 786 patients in a 2-year retrospective study. Five hundred and ninety-four (76 %) died at the scene, and 118 (15 %) died during the course of the hospitalization [5]. Mortality of craniocerebral GSW after admission was 69 % in Aarabi et al.’s study [5] with 30.4 % dead on arrival. Less than 20 % of the total population of craniocerebral GSW will receive neurosurgical treatment [5]. Approximately 50 % of those craniocerebral GSWs who make it to a trauma center alive are discharged to rehabilitation [5].

### Pathophysiology

**Fig. 1:**
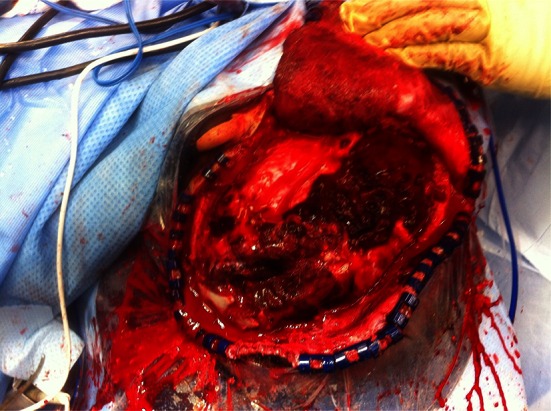
Operative photograph showing a gunshot wound to the left parietal lobe. The craniotomy has been performed and the dura opened. The upper part of the ear lobe is exposed in the upper operative field. *Note* the gross hemorrhagic track of the bullet with surrounding swollen brain. The wound was contaminated with dirt

Ballistic aspects of the wounding should always be considered including the type of weapon used, the proximity of fire, bullet caliber, jacketing and velocity [7, 8]. The volume of injured brain and size of cavitation adjacent to the path of the missile are dependent on the kinetic energy imparted to the brain by the missile. This depends on the velocity of the missile at the point of impact with the head and the thickness of the skull. The extent of brain injury also depends on the size, shape, spin and yaw of the missile, and whether it fragments. The principal pathological effects of craniocerebral GSW are brain swelling, intracranial hemorrhage and penetrating injury with bone and metal fragments and other foreign bodies (see Fig. 1).

### Prehospital care

Maintenance of airway, and adequate ventilation, correction of hypoxia and hypotension are crucial to prevent secondary brain injury. Advanced paramedic training and efficient trauma systems may deliver more craniocerebral GSW victims to trauma centers alive.

### Emergency room treatment (see Table 1)

In a 5-year retrospective review of 132 civilian patients with craniocerebral GSW, increasing survival was associated with aggressive resuscitation in all patients, and resuscitation with blood products and hyperosmolar fluids were independently associated with survival [3]. GCS 3–5 and bihemispheric injury should not prevent early resuscitation, but a decision for expectant supportive care should come when the patient has been stabilized and then reassessed as some may improve. It is therefore the post-resuscitation GCS that should be used for decision making. Excessive crystalloid, permissive hypotension, hypoxia and hypercapnia should all be avoided.

**Table 1:** Emergency room treatment of craniocerebral GSW

Early aggressive resuscitation (‘damage control resuscitation’)
Correct hypotension, hypoxia
Maintain PaCO2 in the normal range
Hypertonic saline for brain swelling
Urgent control of cervical vascular injury
Avoid excessive crystalloid
Early correction of traumatic coagulopathy with combinations of blood, blood products (fresh-frozen plasma, platelets), cryoprecipitate or prothrombin complex concentrate [3]
Surgical airway if gross maxillofacial trauma or facial/respiratory tract burns are present
Packing nasal cavity and facial wounds to control hemorrhage
Urgent CT scan
Tetanus prophylaxis
Antibiotic prophylaxis

Acute traumatic coagulopathy (ATC) may develop in patients with isolated head injury (which includes GSW) and in the setting of multiple injuries with major blood loss and shock [9, 10]. This latter scenario includes multiple gunshot wounds or blast injury. The diagnosis and treatment of acute traumatic coagulopathy should be made rapidly, and replacement of blood and clotting components proceed as soon as possible. Massive transfusion protocols have been developed in many trauma centers [11]. However, the optimal ratio of plasma, packed red cells and platelets to treat acute traumatic coagulopathy is uncertain and remains under investigation [12]. Cryoprecipitate, prothrombin complex concentrates (PCC) and tranexamic acid are variably used in the resuscitation phase [11]. The effect of Vitamin K administration is delayed 6–12 h and so is not useful in the resuscitation phase. Off-label use of recombinant Factor VIIa is an option, but the American Society of Anesthesiologists recommends use on a case-by-case basis because of the risk of serious adverse events [13]. ATC after head injury increases the risk of mortality [14, 15]. Whether early correction of ATC improves outcome remains to be confirmed [11].

### Indications for surgery for craniocerebral GSW

Minor pellet injuries to the brain with small entry wounds may only require local debridement, closure and antibiotics. More severe focal injuries with hemorrhage and fragments without adverse radiological features may also only require local exploration via a small craniotomy. More severe penetrating injuries will require extensive surgery if a decision is made to operate. This may include decompressive craniectomy, debridement, evacuation of hematomas, dural repair and insertion of an ICP monitor. The great challenge and dilemma for the neurosurgeon treating a severe craniocerebral GSW is whether to pursue surgery and survival of the patient at all costs or alternately, whether to pursue quality of survival and therefore expectant treatment in selected patients. The disadvantage of active treatment, including surgery on patients with a predicted poor prognosis, will result in increased numbers of minimal conscious state (vegetative) and severe disability survivors who may be a burden on their family and the healthcare system.

There are a number of clinical findings and imaging features which are significant determinants of outcome (see Tables 2 and 3). These include age, admission GCS, abnormal pupil reactivity, and the trajectory of the missile and obliteration of the basal cisterns [5, 16]. These should all be considered when deciding to pursue aggressive management and surgery on the individual patient [6]. The current management of penetrating injury to CNS is based mainly on retrospective observational studies [17]. Clinical practice guidelines for the management of civilian and military penetrating brain injury have been published [18, 19].

**Table 2:** Clinical factors associated with poor outcome following civilian craniocerebral gunshot wound (GSW) [27, 56]

GCS <5 (post-resuscitation) on admission
Dilated, unreactive pupil(s)
Occipital entry wound
Brainstem injury
Injury to ‘eloquent’ brain
High-velocity missile injury (e.g., semiautomatic military-type weapons)
Hypotension on admission
Major intracranial vascular injury
High ICP
Onset of diabetes insipidus [16]
Suicide attempt (because of close range)
Increased retrieval time
Coagulopathy or disseminated intravascular coagulation (DIC)
Advanced age

Comment: Bilateral frontal lobe injuries (often seen after suicide attempts) have better survival prospects than other bilateral injuries, but cognitive deficits and personality change may be profound

**Table 3:** CT features associated with poor outcome following civilian craniocerebral gunshot wound (GSW)

Multilobar or bihemispheric injury
Ventricular injury with hemorrhage
Diffuse fragmentation
Missile passing through the geographic center of the brain (i.e., involving the thalamus and basal ganglia) An area 4 cm above the dorsum sellae was described as the *zona fatalis* [16]
Trajectory crossing the *x*, *y* and *z* planes
Midline shift >10 mm on CT (Caveat: Kim et al. [16] found midline shift was associated with better outcome presumably because one hemisphere is traversed rather than both hemispheres)
Compressed or obliterated basal cisterns
Large intracerebral hemorrhage
Subarachnoid hemorrhage (SAH) [56]
Large volume of contused brain
Posterior fossa wound with brainstem involvement
‘*Tram track sign*’ hemorrhage on either side of a dark center track in a perforating injury [16] (see Fig. 1)

**Fig. 2:**
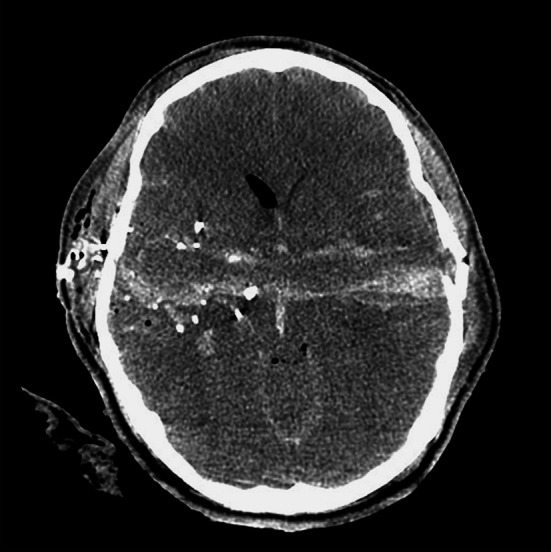
Axial CT scan showing the ‘tram track sign’. The bullet has traversed the cerebral hemispheres with streaks of blood on either side of the low density track. This resulted in a fatal outcome for the patient

Active management has often been withheld in patients with GCS 3–5, particularly if there is a bihemispheric injury. If the trajectory passes through both thalami and basal ganglia or through the posterior fossa and brainstem, the patient is unlikely to survive or be better than vegetative and should be managed expectantly. Kim et al. [16] performed a Cartesian vector analysis on trajectories on CT scans of 217 civilian through-and-through GSW to the head and found midline brain shift was more common in survivors probably because the injury was predominantly unilateral in these cases. They also found that an area of brain approximately 4 cm above the dorsum sellae when penetrated across the midline led to brain death. They coined the term ‘*zona fatalis*’ for this area. Kim et al. [16] also described the ‘*tram track sign*’ which is a dark central track with a hyperdense line of blood on either side (Fig. 1). This sign was associated with fatal injury (*p* = 0.005) (see Fig. 2).

Surgery is not recommended for a craniocerebral GSW, GCS 3 with fixed dilated pupils following resuscitation and no mass lesion on CT [2]. Kaufman et al. [2] recommend operating on patients with GCS 3, if the pupils are reactive and the patient is hemodynamically stable and, for GCS 4–6, if the pupils are reactive and the patients are not hypotensive or if the pupils are fixed and dilated and there is a motor response. Kaufman et al. [2] have reported some good outcomes in these patient groups. This management is clearly controversial, and other clinical and CT scan adverse factors will need to be considered and may sway the surgeon against surgery in these severe craniocerebral GSW. Patients with GSW who suddenly deteriorate with mass lesions on CT should have immediate surgery [2]. In patients with craniocerebral GSW, if there is an intracranial mass lesion, unequal pupils or reacting pupils, we recommend urgent craniotomy unless there is brainstem or bilateral thalamic, basal ganglia injury. Brain swelling with minimal hemorrhage may also be an indication for urgent craniectomy. Each patient should be judged on a case-by-case basis. Based on the current evidence, GCS <5 is not an absolute contraindication for surgery.

### The principles of surgery for craniocerebral GSW

The principles of surgery for craniocerebral GSW have been well described. The salient points are as follows: urgent surgery should be undertaken within 1 h of arrival, preferably within 30 min. A surgical airway should be performed if the upper airway affected by swelling or severe injury. Gaining rapid control of hemorrhage in the brain, head and neck is a vital component of the prevention and treatment of shock and coagulopathy in these patients. Urgent packing of the nasal cavity and facial wounds and balloon tamponade of the nasopharynx may be required to control hemorrhage. Current neurosurgical practice favors more aggressive decompression of the brain (unless there is a focal injury) and less aggressive debridement and retrieval of deep bone and metal fragments [20]. Deep exploration for fragments increases the risk of morbidity. Irrigation of the missile track can release debris. Wide decompressive craniectomy should be performed where there is significant cerebral swelling seen on CT [21]. ICP monitoring is performed. The options are ventriculostomy drain which enables CSF venting and therefore treatment of raised ICP and/or a parenchymal ICP monitor.

The risk of infection increases with acute or delayed CSF leak, paranasal sinus continuity with the cranial cavity, transventricular injury and injuries crossing the midline [22–24] Heavy wound contamination and delayed surgery may also be adverse factors. Therefore, watertight dural closure is essential. The elimination of CSF leak, adequate debridement of contaminated wounds and early surgery are important strategies to reduce infection risk. A duraplasty using pericranium or dural substitute will permit further cerebral expansion and allow for dural closure. Primary scalp closure should be performed. Advancement or rotation scalp flaps may be required to achieve closure.

There is considerable variability in the literature on the use of prophylactic antibiotics [23]. Bayston et al. [25] performed a systematic review of prophylactic antibiotics for penetrating craniocerebral trauma and found only retrospective and anecdotal studies. They recommended broad-spectrum antibiotic prophylaxis for both military and civilian penetrating craniocerebral injury. Staphylococci are important potential pathogens and Gram-negative bacilli may also be involved. Bayston et al. [25] recommended cephalosporin alone or with gentamicin for 5 days as the minimum prophylaxis. When the wounds are contaminated with soil or excreta or where clothing is in-driven, anerobic cover with metronidazole is recommended. The possibility of delayed fungal infection should be also considered [25]. Broad-spectrum antibiotic cover is also recommended in the penetrating brain injury guidelines [23]. Lin et al. [17] recommended broad-spectrum prophylactic antibiotic cover with vancomycin, gentamycin and metronidazole for 48–72 h.

The experience of craniocerebral penetrating trauma in US military personnel from the Iraq and Afghanistan wars is that broad-spectrum cover was associated with appreciable rates of multi-drug resistant organisms particularly Acinetobacter requiring meropenem. Therefore, the current recommendation of the US military guidelines is cephazolin for 5–7 days [26]. We recommend that infectious disease physicians should be involved in the choice and duration of antibiotic prophylaxis for these injuries.

Anticonvulsant prophylaxis is continued for 1 week. Collaboration with ENT, ophthalmology, maxillofacial, plastics and vascular surgeons will be required where there is complex craniofacial wounding. Metal fragments removed at surgery are kept for forensic evidence.

Lin et al. [17] have presented useful practical technical advice to enable rapid and successful surgery. Their mantra for treating GSW to the brain is “time is brain”. Perhaps the remarkable recovery in the high profile case of the near-fatal cerebral GSW injury to Congresswoman Gabrielle Giffords in the USA in 2011 which Lin et al. [17] call the ‘Giffords factor’ will encourage a more aggressive and rapid approach to severe craniocerebral GSW. Giffords had surgery about 38 min from arrival at the hospital. Aarabi et al. [5] performed surgery in 28 of 48 resuscitated patients (58 %). There was simple debridement and skin closure in 9 (19 %) patients, and craniotomy or decompressive craniotomy in 19 (40 %) patients. Of the 5(10 %) who had decompressive craniectomy, 3 were done acutely and 2 for intractable intracranial hypertension.

### The complications of craniocerebral GSW

Complications of craniocerebral penetrating injury have been reviewed [18, 27] and include pseudoaneurysm, cerebral vasospasm, cerebral abscess, meningitis, ventriculitis, epilepsy and hydrocephalus. GSW may cause subarachnoid hemorrhage which is associated with cerebral vasospasm. The vasospasm is diagnosed with daily transcranial Doppler studies. Transluminal angioplasty may be required [28, 29]. Pseudoaneurysms, which have a reported incidence of 20–50 % of penetrating head injuries, require early angiographic diagnosis and definitive multimodality treatment [30]. The criteria for digital subtraction angiography (DSA) following penetrating brain injury have been described by Bell et al. [30]. Those are as follows:

1. Penetrating injury through the pterional/orbitofrontal region
2. Known cerebral vessel injury with or without pseudoaneurysm seen at the initial exploration
3. Blast injury with GCS <8 (closed or penetrating)
4. Transcranial Doppler (TCD) evidence of vasospasm
5. Spontaneous, unexplained decrease in the partial pressure of brain oxygen (PbrO2) [30]

Lead toxicity is uncommon but lead levels should be monitored if there are major embedded metallic fragments. The indications for removal of retained bullet or metal fragments are limited and are outlined in Table 4. Those large fragments located in CSF cisterns or the ventricles should be considered for removal as well as those that are superficially located. Additional indications include heavy metal toxicity symptoms for those in CSF including delayed lead (Pb) and copper (Cu) toxicities. Delayed extraction of a bullet or a major metal fragment may be aided by stereotactic techniques or fluoroscopic ‘c’ arm [31]. Those fragments embedded in brain tissue that do not migrate and are not easily accessible can be followed with noninvasive imaging.

**Table 4:** Criteria for removal of intracranial metal fragments

Large fragments in superficial locations
Heavy metal toxicity
Large fragments within the ventricles
Large fragments within the CSF cisterns
Fragments that are mobile or associated with intermittent hydrocephalus
Fragments/large foreign bodies related to large blood vessels

### The prognosis of craniocerebral GSW

Survival correlates with post-resuscitation GCS, but the figures vary considerably between series (see Table 5), particularly in those with low GCS. Gressot et al. [6] retrospectively reviewed 119 patients admitted to hospital with GSW to the head. The overall outcome was 49 % death, 19 % favorable outcome and 35 % had poor outcome. However, of those with an initial GCS of 3–4, only 11 % had a good outcome and 89 % had a poor outcome or death, whereas for those with GCS 5–15, there were 27 % good outcomes and 73 % poor outcome or death. In Aarabi et al.’s [5] series, 20 patients were admitted with GCS 3–5 and 19 (95 %) of those died. Of 8 patients with GCS 6–8, one died, one was severely disabled and 3 had mild to moderate disability. All 4 patients with GCS 9–12 had Glasgow Outcome Score (GOS) of 4 which is a moderate disability (disabled but independent). Of 13 patients with GCS 13–15: 2 died, 7 had GOS 4 and 4 were lost to follow-up. Lin et al. [17] report on 4 patients with GCS <5 on admission with civilian craniocerebral GSW. Two had a unilaterally dilated pupil, 2 had equal and reacting pupils. One had a GCS 3. Three out of 4 were functionally independent at 1 year. Joseph et al. [3] reported survival of 28 % in patients with GCS 3–5 and 22 % in patients with bihemispheric injuries. Of those who presented with a GCS 3–5, 18 % were discharged with a GCS >8. The proportion who became independent is unknown. Glapa et al. [32] reported a series of 72 civilian patients with a mortality of 81 % for GCS ≤8 versus 14 % for GCS >8.

**Table 5:** Survival for civilian GSW to the head [27] [57]

GCS after resuscitation	Survival
GCS 3–5	0–8.1 % [27], 28 % [3], 100 % in 4 patients [17], 0 % for GCS 3, 4 [16], 5 % [5], 40 % (GCS 3–4) [6]
GCS 6–8	35.6 % [27], 83.3 % (with 2 lost to F/U) [5]
GCS 9–15	90.5 % [27], 84.6 % (with 4 lost to F/U) [5]

There are many clinical factors associated with poor outcome following civilian craniocerebral GSW. These are outlined in Table 2 and include the time to reach a neurosurgeon, age, GCS post-resuscitation, pupil size and reactivity, and the presence of hypoxia or hypotension. GCS >8 is one of the most important predictive factors for a good outcome [5]. The weapon ballistics should also be considered by the clinician.

Certain CT features are associated with poor outcome following civilian craniocerebral GSW and are outlined in Table 2. The trajectory of the bullet in crossing ‘*x*’, ’*y*’ and ‘*z*’ planes was more significant on regression analysis than obliteration of basal cisterns and intraventricular hemorrhage [5].

## Blast injury to the brain

Blast injuries due to improvised explosive devices (IEDs) have been increasingly encountered in the Iraq and Afghanistan wars and in terrorist events in many countries. The pathophysiology of blast injury is more complex than GSW [33]. Bomb explosions cause injury to the brain by three main mechanisms: (1) the overpressure wave which is transmitted through the skull and is also probably ‘funnelled’ through skull openings (orbits, nasal cavity, temporal bones and foramen magnum; (2) metal fragments and other foreign bodies penetrating the skull and entering the brain; (3) hot gases generated by the blast cause skin and respiratory burns [33].

**Fig. 3:**
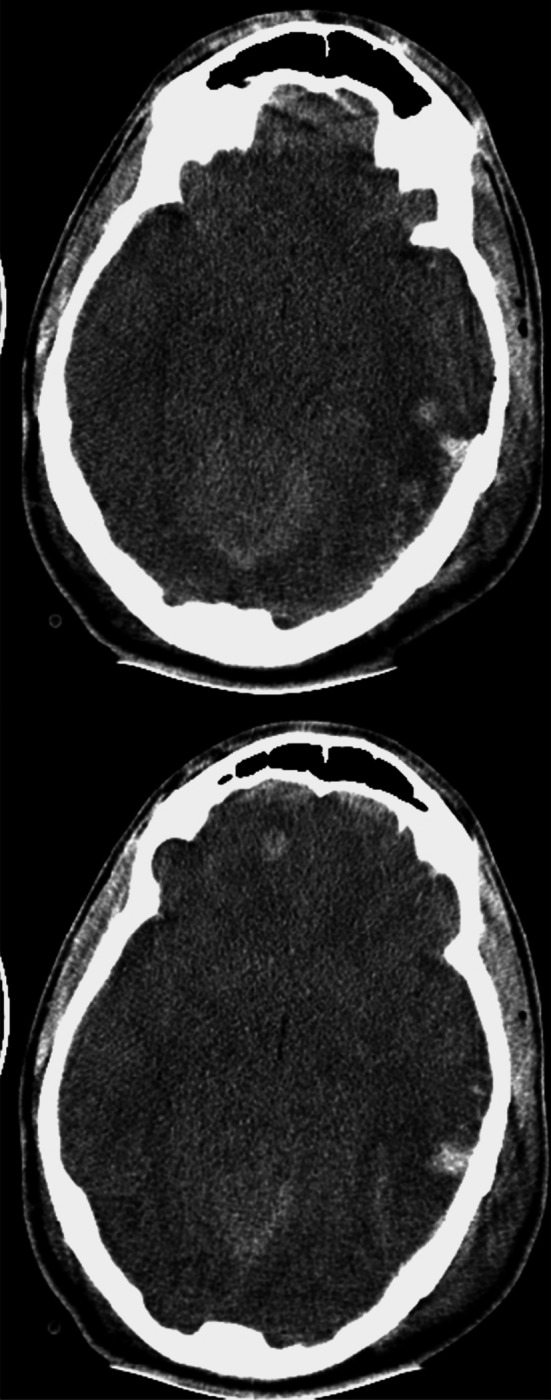
Axial CT scans showing non-penetrating blast overpressure effect on the brain. *Note* the generally swollen brain with loss of basal cisterns, slit-lke third ventricle, loss of *gray-white* differentiation and multiple hemorrhagic contusions

The blast wave frequently causes severe cerebral edema (see Fig. 3). Moderate and severe blast injuries frequently involve penetrating craniocerebral injury and are usually a component of polytrauma rather than isolated head injury. These wounds are usually heavily contaminated. Subarachnoid hemorrhage is common. Civilian neurosurgeons should become familiar with the patterns of blast injury and the management.

### Principles of management

**Fig. 4:**
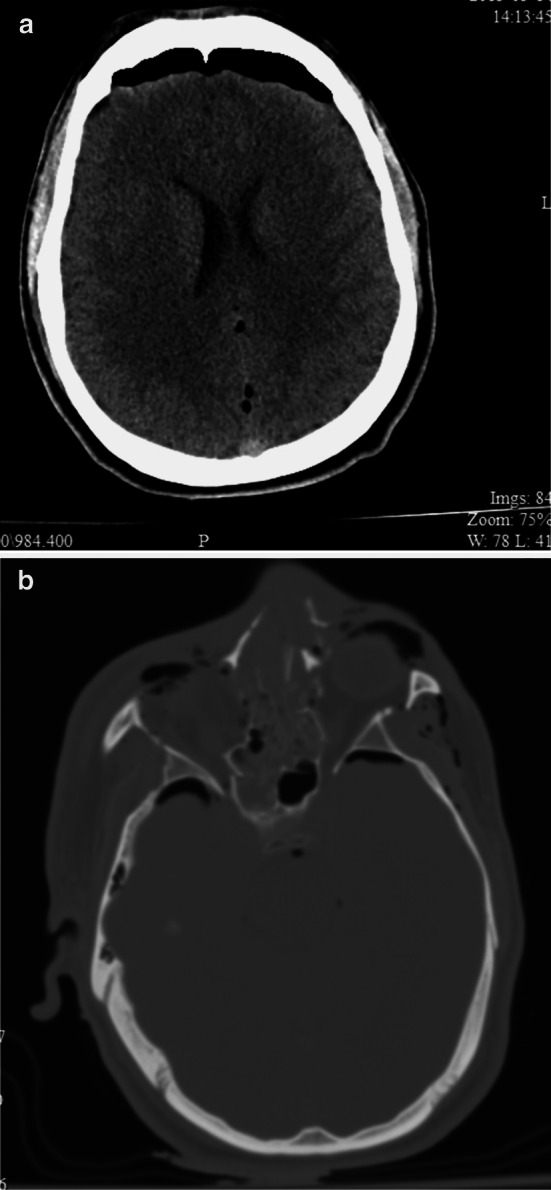
**a** IED injury in a 23-year-old male causing facial lacerations, right globe disruption, oral lacerations, penetrating neck wound and lower extremity injuries. He had fractures to the midface, right orbit and frontal sinus. Axial CT showing a large frontal aerocele and multiple other small intracranial air bubbles. **b** Axial CT bone windows showing extensive fractures to the nasal bones, ethmoid and sphenoid sinuses and the right orbit

Multidisciplinary teams are best-equipped to manage these complex injuries of the head and neck, and the treatment has recently been reviewed [33]. A thorough primary and secondary survey are mandatory. The external wounds can be deceptive and do not reveal the extent of internal damage or the trajectory and final position of penetrating fragments. Facial and sinus penetration, orbital injury and skull base disruption are often present given the upward and outward trajectory of blasted fragments (see Fig. 4a, b). With these injury patterns in mind, management principles are guided by rapid cranial decompression [21], early repair of skull base injury with consideration of CSF diversion, early diagnosis and management of traumatic vascular injuries which are common, and delayed facial and cranial reconstruction to allow for resolution of the inevitable local and systemic infections that arise.

Hemicraniectomy also protects patients from the effects of brain swelling during air transport to definitive care [34]. The problem of SAH, vasospasm and pseudoaneurysm described in the craniocerebral GSW section also occurs following blast injury [28–30].

### Outcome

In the prospective study of Weisbrod et al. [35], 32 % of those presenting with a GCS 3–5 and 63 % of those with GCS 6–8 achieved functional independence 2 years following severe blast or penetrating TBI. Significant improvement may occur in individuals with severe blast or penetrating TBI over one to 2 years [35, 36].

In a retrospective study of 604 patients, outcomes of a military population with isolated blast and penetrating severe TBI compared favorably with those of a matched civilian population [37]. A lower overall mortality was found in the military population (7.7 vs. 21.0 %; *p* < 0.001; odds ratio 0.32 [0.16–0.61]). This difference was more pronounced in the penetrating group (5.6 vs. 47.9 %; *p* < 0.001; odds ratio, 0.07 [0.02–0.20]) [37]. Many factors may account for the differences, including higher neurosurgical intervention rates and fewer high-velocity single-bullet injuries in the military population [33].

## Craniocerebral stab wounds

Craniocerebral penetrating injuries due to knives, arrows, nail guns, spears and other sharp implements are uncommon. Machete injuries to the head are common in parts of Africa. These may cause compound depressed fractures with neurological deficit [38]. The principles of surgery for craniocerebral stab wounds are the same as for GSW. The foreign body should not be removed without surgical control of major vessels in proximity. Most craniocerebral injuries in children are due to sharp objects penetrating the orbit. These may initially appear trivial and are often missed if the foreign body is withdrawn. The child may present with delayed infection including frontal lobe abscess [25].

## Spinal GSW

GSW to the spine have been extensively reviewed [4, 27, 39–43].

### Epidemiology

GSW to the spine accounts for 13–17 % of all gunshot injuries and occurs predominantly in the thoracic region in civilian practice [4, 44]. Penetrating injury accounts for about half of all spinal cord (SC) injuries in urban centers [45]. The rate of complete SC injury in cervical GSW is about 70 %, and the rate of incomplete injury in lumbosacral injuries is about 70 % [4].

In a recent series of military spine injury, cervical spine was the most common but this included closed injuries [39].

Schoenfeld et al. [46, 47] documented a spine casualty rate of 7.4 % in a cohort of 4,122 soldiers deployed to a combat zone in Iraq and an 11.1 % rate of spinal injuries in a retrospective study of 7,877 combat wounded from Iraq and Afghanistan recorded in the Defence Trauma Registry 2005–2009. These are the highest figures recorded in US military history although blunt injury is also included. The incidence of combat-related spinal trauma was 4.4 per 10,000 [47]. In a series of 701 soldiers injured in Iraq and Afghanistan, SC injury occurred in 12 % of all casualties and represented 4 % of all musculoskeletal wounds [48]. This is thought to be due to the increased use of improvised explosive devices (IEDs). In a series of 90 British military casualties with penetrating neck injury, 20 (22 %) had cervical spine or spinal cord injury. Only 6 (7 %) of these survived to reach hospital and 4 of the 6 subsequently died within 72 h [49]. Spinal injuries in combat troops are frequently accompanied by adjacent visceral injuries and limb injuries [50]. Blair et al. [51] reported that 28 % of US military spine casualties had isolated penetrating injuries, 66 % had isolated blunt injuries and 5 % had a combination of both.

### Pathophysiology

The extent of injury to the spinal cord depends on ballistics, the degree of transection and contusion of the SC, the degree of concussive blast injury of the SC, compression of the cord by hematoma or displaced bone fragments, disruption of SC vasculature and the mechanical stability of the spinal segment(s) involved.

### Principles of management of penetrating spinal injury

Acute management includes detailed documentation of neurological status, maintenance of adequate spinal cord oxygenation and perfusion. Exploration of the spine in urban civilian injury is not usually required because the deficit is not usually improved by surgery and there is usually no mechanical stability. However, there is an increased risk of mechanical instability in patients with cervical GSW causing SC injury [52]. A hard cervical collar should be applied until CT or MR is obtained for spine clearance [46]; however, where there is penetrating injury, spinal precautions or application of hard cervical collar should not hinder the management of the acute neck injury and should be re-applied when these procedures are completed [52]. Spinal canal surgical decompression may create instability.

Contaminated wounds should be irrigated and debrided. Injuries to adjacent structures in the neck, torso and pelvis relate to the trajectory of the missile and will require the relevant investigation and treatment. Bullets passing through the gastrointestinal tract risk causing sepsis as they enter the spine. There is scant evidence on the type and duration of antibiotic prophylaxis in penetrating spine trauma. Recommendations vary in the literature from 2 to 10 days [41, 43]. We recommend a minimum 2 days of broad-spectrum antibiotic cover, but would increase the duration if there is bowel content contamination. Steroids are not indicated and may increase the risk of non-spinal complications [4, 41].

Partial SC injury or nerve root injury due to compression by bone, metal fragment or hematoma may benefit from decompression [43]. Surgical decompression of intracanal bullets involving lumbosacral spine, with incomplete deficit and cauda equina syndrome, may result in motor and sensory improvement [4, 41]. Minimally invasive surgical techniques may be used in selected cases [44, 53].

Surgery is also indicated for complications such as infection and mechanical instability. We recommend repair of persistent external CSF fistulae [45]. The risk of infection including meningitis increases if CSF fistula persists. Migration of metal fragments and late lead poisoning are both uncommon so that preventive surgery is not necessary.

GSW to the atlantoaxial spine is uncommon and often fatal. Ten cases were recently reported by Syre et al. [54]. Unilateral injuries were usually stable and did not need surgery. Unilateral vertebral artery injury is usually well tolerated and vascular complications can be managed with endovascular techniques. Only one patient required fusion for stabilization [54].

### Outcome

The outcome is determined primarily by the level of the spinal injury and the severity of neurological deficits rather than the method of treatment.

A US study of 60 adolescents with GSW to the spine included 34 patients with complete neurological deficit [45]. No patient required surgery. At 1 year follow-up, there was no spinal instability and there was significant but non-functional improvement [45]. Improvement of fixed neurological deficit is uncommon. In a retrospective series from New Orleans, Trahan et al. [53] reported 127 (88 %) patents treated conservatively, and only one (0.7 %) improved from ASIA D to E. Of 20 patients who underwent surgery, one (5 %) patient had a clinical improvement from ASIA C to D. Sidhu et al. [4] performed a systematic review of civilian GSW to the spine and found patients in the non-operated group with partial SC injuries had a weighted rate of neurologic recovery of 65.3 and 12.7 % of complete lesions improved, whereas in the operated group, these figures were 53 and 21.5 %, respectively. Sidhu et al. [4] conclude that there is no major benefit for improvement in neurological deficit with surgery. The rate of complications is greater in the operated group, but there is a bias here because these patients may have more severe injuries that require the surgery.

## Blast injury to the spine

Blast injuries and high-velocity GSW to the spine which are encountered by military surgeons tend to be more destructive and require exploration more readily than civilian GSW. Internal fixation for instability is more often required. Blast injuries to the spine with penetrating fragments are usually heavily contaminated and require debridement. Lumbar burst fractures and lumbosacral dissociation may occur in spinal blast injury [39]. Military surgeons recommend decompression for an incomplete neurological injury and continued canal compromise, within 24–48 h of injury with stabilization if there is spinal instability [43].

## Spinal stab wounds

Penetrating spinal injuries due to knives or other sharp objects are rare in most settings. The lower cervical and thoracic regions are most commonly affected due to assaults from behind. The management has been well described by Shahlaie et al. [55]. Removal of the foreign body may be beneficial both acutely and in cases of delayed presentation.

## Conclusions

Craniocerebral GSW is frequently a devastating injury with 66–90 % of victims dying before they reach hospital and up to 51 % of those treated in hospital surviving. The decision to operate depends on many factors including GCS, age, pupil size and reaction, ballistics and imaging features on CT scan. Once this decision has been made, urgent surgery follows. Improved outcome has been reported in recent series probably because of the rapidity of resuscitation, correction of coagulopathy and surgery. The principles of management of cranial blast injury are similar to GSW. Penetrating spinal injury does not usually require exploration unless the injury is unstable or there is a compression with partial spinal cord or cauda equina injury or complications develop. Multidisciplinary teams including experienced clinicians treating patients with penetrating CNS injury is likely to produce the best outcomes.
